# Diagnosis and management of children with McArdle Syndrome (GSD V) in New South Wales

**DOI:** 10.1002/jmd2.12389

**Published:** 2023-08-09

**Authors:** Louisa Adams, Arthavan Selvanathan, Kiera J. Batten, Nancy van Doorn, Susan Thompson, Ashleigh Mitchell, Hugo Sampaio, Troy Dalkeith, Jacqui Russell, Carolyn J. Ellaway, Michelle Farrar, Carolyn Broderick, Kaustuv Bhattacharya

**Affiliations:** ^1^ Genetic Metabolic Disorders Service Sydney Children's Hospitals' Network (Randwick and Westmead) Sydney Australia; ^2^ School of Health Sciences University of New South Wales Sydney Australia; ^3^ Children's Institute of Sports Medicine Children's Hospital at Westmead Westmead Australia; ^4^ Faculty of Medicine and Health, Westmead Campus University of Sydney Westmead Australia; ^5^ Discipline of Paediatrics, School of Women's and Children's Health UNSW Medicine Sydney Australia; ^6^ Department of Neurology Sydney Children's Hospital Randwick Randwick Australia

**Keywords:** aerobic, exercise, glycogen storage, McArdle, rhabdomyolysis, sports physiology, VO_2_ max

## Abstract

Glycogen storage type V (GSD V—McArdle Syndrome) is a rare neuromuscular disorder characterised by severe pain early after the onset of physical activity. A recent series indicated a diagnostic delay of 29 years; hence reports of children affected by the disorder are uncommon (Lucia et al., 2021, *Neuromuscul Disord*, 31, 1296–1310). This paper presents eight patients with a median onset age of 5.5 years and diagnosis of 9.5 years. Six patients had episodes of rhabdomyolysis with creatine kinase elevations >50 000 IU/L. Most episodes occurred in relation to eccentric non‐predicted activities rather than regular exercise. One of the patients performed a non‐ischaemic forearm test. One patient was diagnosed subsequent to a skeletal muscle biopsy, and all had confirmatory molecular genetic diagnosis. Three were homozygous for the common *PYGM*:c.148C > T (p.Arg50*) variant. All but one patient had truncating variants. All patients were managed with structured exercise testing to help them identify ‘second‐wind’, and plan an exercise regimen. In addition all also had an exercise test with 25 g maltodextrin which had statistically significant effect on ameliorating ratings of perceived exertion. GSD V is under‐recognised in paediatric practice. Genetic testing can readily diagnose the condition. Careful identification of second‐wind symptomatology during exercise with the assistance of a multi‐disciplinary team, allows children to manage activities and tolerate exercise. Maltodextrin can be used for structured exercise, but excessive utilisation may lead to weight gain. Early intervention and education may improve outcomes into adult life.

**Synopsis**

Early diagnosis and intervention using serial exercise physiology assessments in McArdle Syndrome patients can allow children prospectively to manage their condition effectively.

## 1 INTRODUCTION

Glycogen storage type V (GSD V—McArdle Syndrome) is a rare inborn error of metabolism affecting catabolism of glycogen within skeletal muscle.^1^ It is an autosomal recessive disorder of muscle glucose metabolism (MIM#232600) and is caused by mutations in the *PYGM* gene on chromosome 11q13, which encodes the muscle isoform of glycogen phosphorylase.^2^, ^3^ It is clinically characterised by exercise intolerance, muscle cramps and a risk of severe rhabdomyolysis. In some instances, this can lead to compartment syndrome leading to decompressive fasciotomy.^4^ The enzymatic deficiency results in the failure of the breakdown of muscle glycogen to glucose‐1‐phosphate.^5^

Unique to GSD V is the ‘second wind phenomenon’ first described by Pearson in 1961.^6^ This describes a noticeable reduction in exercise induced pain after a short period of time, with corresponding decrease in heart rate (HR) and breathing effort. The time to second wind varies in the literature, with adult studies reporting it occurring as early as 4 min and up to as late as 15 min, most commonly occurring around 7 min.^7^, ^8^ Use of pre‐exercise glucose and sucrose has been demonstrated to ameliorate pain and physiological parameters in several cohorts of adult patients.^9^, ^10^, ^11^ We have elected to use maltodextrin, which is an oligosaccharide of up to 10 glucose molecules instead, because the same products can be used for all relevant glycogen storage and fat oxidation disorders, that our team manages, in the acute setting. It can also be medically prescribed in a school setting. Various other treatments have not yet, demonstrated consistent benefit.^12^, ^13^, ^14^, ^15^, ^16^, ^17^, ^18^ GSD V is considered a rare disorder, with an estimated population prevalence between 1:100 000 and 1:167 000.^19^, ^20^

Median diagnostic delay has been reported to be 29 years.^1^, ^20^ There are consequently scant reports characterising paediatric manifestations and management.^21^ Whilst exercise related symptoms are typically experienced in childhood, children may not present clinically for assessment due to exercise avoidance.^22^ This can set in train behaviour that avoids exercise or exertion which can lead to life‐long difficulties, including exclusion from social activities, deconditioning and lower quality of life compared to the normal population.^23^, ^24^ Having identified, several cases in our service, we believe that children with GSD V need to be identified, such that children can recognise and manage their condition prospectively. Exercise interventions studies in adults with GSD V suggest that exercise training is safe and effective with the potential to improve aerobic fitness and muscle strength.^25^ Greater awareness is required amongst paediatricians and paediatric neurologists about the specific activities tolerated by this group of individuals. The purpose of this study was to identify the presenting features of children with GSD V and subsequent management. This includes diagnostic molecular genetic testing data and exercise physiology data with and without maltodextrin. Subsequent hospitalisation acute management plans and prospective exercise plans are presented, leading to a comprehensive report on the management of children with GSD V.

## 2 METHODS

Retrospective chart review from a single state‐wide centre in New South Wales, Australia. Data were collected on clinical presentation, exercise testing, genetic diagnosis, acute illnesses requiring hospital admission, exercise management plan until 31 December 2022. Descriptive analyses for non‐parametric data used median values and range. Statistical analyses were performed using GraphPad Prism (version 9.4.1—La Jolla, California). Paired data for HR, pain and rating of perceived exertion (RPE), using the Borg scale, were assessed with, and without 25 g maltodextrin pre‐test, for 12 min (by which time all patients had exhibited second‐wind physiology). Area under the curve for the whole exercise test, for the above three variables, was compared between the two groups. A paired *t*‐test was used to analyse data, assuming parametric distribution of variables, two‐tailed distribution and statistical significance of *p* < 0.05.

## 3 RESULTS

A total of eight children were identified with a confirmed genetic diagnosis of GSD V, five males. There was one set of identical twins in the cohort (patients 2 & 3). One of the patients had an older sibling with GSD V, managed by an adult service (patient 6). The age at diagnosis ranged between 6 and 16 years of age with a median of 9.5 years. The majority of patients reported a significant history of muscle cramps, poor exercise tolerance and pain since a young age. None of the patients or carers identified that pain improved after a period of time, hence did not identify the second wind phenomenon. A total of six patients had a history of significant rhabdomyolysis, with plasma CK >50 000 U/L (<235) at presentation. In four of these cases, rhabdomyolysis with hospital admission, did not lead to immediate consideration of GSD V being the aetiology.

The time between first reported symptoms and diagnosis ranged from 1 to 15 years, with a median of 5.5 years. The longest delay was approximately 15 years, in a patient who had exhibited symptoms of poor exercise tolerance since infancy and was diagnosed molecularly based on a neuromuscular panel aged 16 years after an episode of significant rhabdomyolysis. The time to diagnosis in patient 6 was less than a year. She presented with an episode of significant rhabdomyolysis and was diagnosed with a subsequent neuromuscular gene panel, on the background of her older sister also having a raised CK and exercise limitation. Both were subsequently confirmed as having GSD V. Patient's 7's time to diagnosis was also only 1 year, however it should be noted that they she too had an older sibling with GSD V who took 14 years to receive a diagnosis, hence family history led to earlier diagnosis in both case six and seven.

### 3.1 Investigations

All patients were diagnosed based on genetics studies, most commonly using a neuromuscular gene panel.

Various other investigations performed across the cohort included acyl carnitine profile, CPT II enzyme assay, dystrophin MLPA and echocardiogram. Patient 5 had a muscle biopsy which was PAS negative but had absent myophosphorylase. Patient 6 had an older sister who had previously had a muscle biopsy which did not note increased glycogen. Patient 6 had an episode of rhabdomyolysis at age 6 years leading to the supervising neurologist performing a non‐ischaemic forearm test, specific genetic testing of both siblings for GSD V and re‐analysis of the muscle biopsy in the older sibling. This too showed normal PAS staining and absence of myophosphorylase. Muscle biopsies are no longer routinely performed, but if they are done, may not show glycogenosis as previously reported.^26^

### 3.2 Molecular characteristics

All patients had confirmed biallelic variants in the *PYGM* gene, aside from the identical twin of patient 2 (he had clinical features of GSD V and hence his genotype was assumed). Of those who had molecular testing, three were homozygous for the common *PYGM*:c.148C > T (p.Arg50*) variant, and another three had this variant in trans with another pathogenic variant (c.613G > A, c.373del and c.253_254insTA, respectively). Patient 6 had a homozygous canonical splice variant, c.1403 + 1G > A, which has been previously reported in a patient with clinical features of McArdle's and absence of phosphorylase staining in muscle.^27^

Only one variant detected in patients in our case series was non‐truncating (c.613G > A causing p.Gly205Ser). This is the second most frequent variant reported in Caucasian populations, and has functional evidence to confirm pathogenicity that are discussed in more detail below.^28^

### 3.3 Exercise data

Seven of eight patients underwent formal exercise testing using gas analysis with the Ultimata metabolic cart on either the cycle ergometer or treadmill. Fixed load and graded exercise tests were performed to a maximum of 60% predicted VO_2_ max.^29^, ^30^ Measures recorded included VO_2_, baseline and maximum HR, rating of perceived pain (on a 10‐point scale), rating of perceived exertion (on a 10‐point scale) and presence (and timing) of second wind phenomenon. Testing was repeated using the same load within a 2 month time frame with an oral 25 g maltodextrin load and the same data were recorded including baseline and max HR, pain scale and presence of second wind.

Patient 7 inadvertently underwent testing using a Bruce incremental protocol and thus was excluded from the analysis, but data are presented in Figure 2. All subjects are reviewed regularly by the multi‐disciplinary team with examples of additional tailored exercise studies provided in Figure 2.

## 4 RESULTS OF EXERCISE TESTING

**FIGURE 1:**
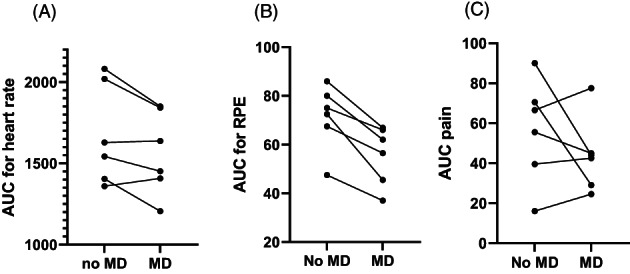
Area under curve (AUC) for exercise test for with and without maltodextrin (MD) for six patients using the same sub‐maximal exercise test for parameters (A) heart rate (HR) (*p* = 0.10); (B) rating of perceived exertion (RPE; *p* < 0.05), using modified Borg score and (C) pain score (0–10) with 10 being most severe (*p* = 0.27). Student paired *t*‐test for statistical analysis.

All patients who underwent exercise testing demonstrated a ‘second wind’. This occurred between 6 and 10 min mark for all patients. Onset of pain occurred between 1 and 7 min mark for all patients. Patients were tested with and without 25 g maltodextrin administered 5 min before the test (Polyjoule—Nutricia‐Danone, Paris, France or CarbPLus—Flavour Creations, Australia). Area under curve was calculated for the duration of each profile, per individual, for HR, RPE and pain score with individual data indicated in Figure 1. With the addition of maltodextrin, HR appeared to decrease in most patients but was not statistically significant (*p* = 0.10), but RPE decreased in all patients (*p* = 0.002) and there was no statistical difference for pain (*p* = 0.2).

## 5 CLINICAL EPISODES AFTER DIAGNOSIS

Five patients had hospital admissions due to rhabdomyolysis after diagnosis (Table 1). These were particularly precipitated by isometric and plyometric exercises that patients were unaccustomed to doing. These included Leapfrog (jumping over poles), hip hop dancing, running up bales of hay, running up several flights of stairs to attend pop concert, jumping off a boat and treading water, downhill skiing and performing a plank exercise. These all resulted in lower limb and back pain. In one subject, shoulder and abdominal pain occurred after cardiopulmonary respiratory resuscitation during basic life support training and arm pain after a paper plane competition. The episodes of rhabdomyolysis were generally associated with extremely high elevations of CK (>50 000 u/L). All were treated with hyperhydration and there was no evidence of renal impairment, either by urine output or by assessment of contemporary renal function.

**TABLE 1:** Indicates diagnostic parameters of children with GSD V, who they were referred to and genetic variants of PYGM and subsequent hospital admissions.

Case	Age of onset (yrs)	Age at diagnosis Yrs	Current age (yrs)	Presenting symptoms	Initially referral to	Initial Investigations	How diagnosed	No. admissions	Genotype
1	4	9	14	Proximal exertional lower limb weakness—climbing up stairs or walking up incline. Stops and rests for a couple of minutes then able to continue on.	Paediatric neurologist age 9	LFT, electrolytes, FBC, TFT, CPTII Acylcarnitine profile lactate, glucose, ketones, CGH microarray, urine organic acids dystrophin MLPA, Pompe Echo cardiogram normal	Neuromuscular panel	0	Homozygous *PYGM*:c.148C > T (causing p.Arg50*)
2	7	8	16	Recurrent rhabdomyolysis. CK of 92 000 after playing on bike. CK of 49 000 after sprinting. CK of 60 000 after being on water slide.	Paediatrician then paediatric neurologist.	Echocardiogram normal, dystrophin MLPA negative	Neuromusuclar panel	5	*PYGM*: c.148C > T (causing p.Arg50*) and c.613G > A (causing p.Gly205Ser)
3	5	8	16	Rhabdomyolysis aged 5. CK of 140 000. Persistent significant elevations of CK after exercise	Paediatrician then paediatric neurologist.	Echocardiogram normal, dystrophin MLPA negative	Neuromusuclar panel	9	Genotype assumed (identical twins).
4	10	16	20	Rhabdomyolysis with peak CK of 154, 000. Symptoms since aged 10 years. Vomiting and poor exercise tolerance at school, climbing hills. OK with dance classes. Deconditioned with poor exercise tolerance at time of diagnosis.	Paediatrician then Metabolic physician after severe rhabdomyolysis.	Cardiology review normal for poor exercise tolerance	GSD/Rhabdomyolysis panel	2	Homozygous *PYGM*: c.148C > T (causing p.Arg50*)
5	1.5	8	19	Delayed motor skills as a toddler. Eventually caught up with development. Leg pain on exertion. Leg pain after running around sports field, stop for 5–10 min. After resuming would be ok.	Paediatrician. Then Paediatric neurologist aged 18 months. Muscle biopsy aged 8	CK elevation at baseline—800, normal UMS, hip Xray and thigh USS. Normal cardiac echo aged 8 years. Muscle biopsy aged 8 years—inconclusive PAS staining and absent myophosphorylase	Muscle biopsy suggestive. Follow up with McArdle variants	3	Compound heterozygous *PYGM*: c.148C > T (causing p.Arg50*) and c.373del (causing Glu125Lysfs*170)
6	6	6	16	Rhabdomyolysis, CK of 72 000 after an athletics day at school. Pain in the middle of 100 m run, slowed down and the pain resolved so kept running. CK after 4–5 h demonstrated 72 000.	Paediatric neurologist (neuromuscular) after rhabdomyolysis episode.	Acylcarnitine, lactate, organic acids ammonia, non‐ischaemic forearm test positive Muscle biopsy in older sibling reviewed and glycogen phosphorylase staining requested ‐ type 1 fibre hypotrophy, absence of myophosphorylase	Clinical presentation, non‐ischaemic forearm exercise test then targeted genetic testing 2013	0	Homozygous *PYGM*: c.1403 + 1G > A
7	5	7	12	Calf pain after walking up hills. After 30 s rest, kept going. Pain on flat surfaces after walking. Diagnosed based on sister proband. Sister had a long diagnostic journey with symptoms aged 5 years, rhabdomyolysis aged 12 years, slow running in races at school. Second wind recognised in sibling.	Paediatric neurologist	Acid alpha glucosidase normal	Neuromuscular Genetic panel	0	Compound heterozygous *PYGM*: c.148C > T (causing p.Arg50*) and c.253_254insTA (causing p.Tyr85fs*5)
8	1.5	16	18	Calf and knee pain walking up hills, needs to stop and rest for 20–30 s, cannot keep up with team in rugby/basketball. Rhabdomyolysis in June 2019 CK 170000 after weight, basketball and swimming session	Paediatric neurologist/neuromuscular service.	Dystrophin MLPA negative, baseline CK 1986, ECG normal	Neuromuscular panel	1	Homozygous *PYGM*: c.148C > T (causing p.Arg50*)

The participation in regular exercise and physical activity varied between individuals. One individual was regularly able to play netball for the school team, and another badminton. Three individuals regularly play football and most are able to go swimming after structured warm up exercises. For competitive structured exercise, children were recommended pre‐exercise supplementation with maltodextrin and most used this regimen. The majority of children felt pre‐exercise carbohydrate supplementation, combined with warm up strategies helped to alleviate symptoms.

Regarding comorbidities of the cohort, two patients had ADHD and were on medication. After the diagnosis of ADHD and institution of medication the frequency of rhabdomyolysis decreased for these two patients.

## 6 DISCUSSION

This study shows the longitudinal diagnosis and management of children with GSD V. In the last 10 years, patients have been identified by a neuromuscular molecular diagnostic panel typically after myopathy and elevated CK or after an acute event of rhabdomyolysis. The second wind phenomenon was not elicited by clinical history to any of the physicians the patients presented to. After referral to metabolic physicians, the second wind was demonstrated to the children and families by our multi‐disciplinary team. Most had ameliorated symptoms after taking maltodextrin and participated in structured exercise, managing this by utilising the second wind. Whilst previous adult studies have used 30–75 g sucrose to demonstrate clinical improvement, we chose to use 25 g maltodextrin or glucose polymer, allowing for body size, likely better tolerance and rapid source of glucose.^10^, ^11^, ^20^, ^31^, ^32^ The patients demonstrated reduced RPE and a trend towards reduced HR but no consistent effect on pain. Rating of pain in this condition can be difficult as some report pain at rest, and children can be unreliable historians. This is a small study and data are preliminary. It appears that subjects that exerted themselves the most, had the most pain, which was ameliorated by maltodextrin.

**FIGURE 2:**
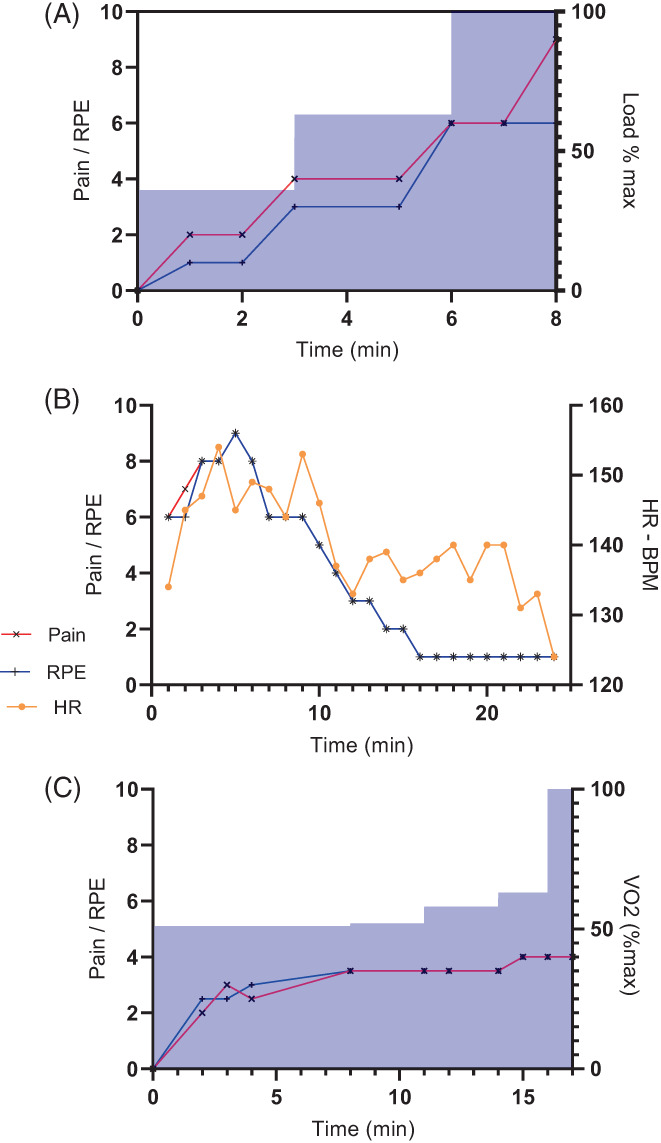
Exercise tests performed in three different teenagers, in different circumstances in McArdle syndrome (A) inadvertent Bruce protocol exercise test with incremental load on treadmill—shaded area to 100%—indicating rapid increase in pain and RPE as load increases. (B) 60% submaximal treadmill test indicating patient was able to continue exercise for 24 min with reduced pain and RPE. (C) Cycle exercise test monitoring gas exchange demonstrating increase load is tolerated after second wind has occurred. HR, heart rate; RPE, rating of perceived exertion.

Our experience managing our first child with GSD V led to the individual taking maltodextrin up to five times a week providing an additional 800 kcals per week with concomitant weight gain from the 75th percentile to 90th percentile. We have used maltodextrin as a medically prescribed supplement which has made use in a school environment acceptable. The use of sucrose may encounter more difficulty. We have subsequently used exercise training, utilisation of the second wind phenomenon through an extended warm‐up and symptom recognition as the predominant intervention in these children, despite the improvement in physiology with maltodextrin. Figure 2A indicates how intense increase in exercise load such as using a Bruce protocol leads to concomitant increase in pain and RPE. This may account for the variation in timing of second wind in different publications as they are fundamentally linked to load, which elicits aerobic phosphorylation. In this subject using the same protocol with maltodextrin did not improve RPE, pain or duration of exercise (data not shown). However, in other patients, duration of exercise has been extended with the same load (Figure 2B) or an incremental load protocol (2c) if they occur after the second wind. Hence, initial exercise testing regimen needs to be sub‐maximal; we currently utilise 60% of predicted VO_2_ max. Using this as our testing regimen, the median time of pain reduction was 10 min (range 8–13 min). Having patients understand their own physiology at a young age may lead to improved secondary gains of sustained exercise.^25^, ^33^, ^34^

Despite management of regular exercise, certain activities provide unexpected demands on the muscle leading to rhabdomyolysis. These particularly include isometric exercise (tonic contraction), for example performing a ‘plank’, or plyometric exercise (rapid stretching and contracting of muscles), for example burpees or hip‐hop dancing. This is well recognised in adult cases, so our management involved teaching children to avoid these situations.^35^

Contrary to adult literature, only one of these patients performed ischaemic or non‐ischaemic forearm testing.^36^ The test is not routine in paediatric practise, requires compliance of behalf of the child and carries some risk of potential rhabdomyolysis and even compartment syndrome.^36^, ^37^, ^38^ Conversely exercise testing allowed the team to gauge the child's aerobic fitness, demonstrate second wind and teach children that they could perform prolonged exercise, utilising aerobic metabolism. Sequential testing allows patients to appreciate the improvements they have made.

None of the patients experienced renal impairment during a rhabdomyolysis event. It would be our normal practise to admit patients with significant pain, myoglobinuria or gross elevation of CK to hospital for hyper‐hydration. Intravenous fluids are administered at 1.5 to 2 times maintenance fluid requirement. With undiagnosed rhabdomyolysis, the fluids would normally be normal saline with 10% dextrose mainly to manage fat oxidation disorders. However in GSD V, the insult occurs due to energy insufficiency early on in exercise and there is no evidence that increased energy delivery is required when exercise has ceased and tissue injury is in‐train.

There has been no genotype–phenotype correlation identified in GSD V to date.^19^, ^39^, ^40^ This is because almost all variants, including those that are non‐truncating, result in absence of myophosphorylase staining on muscle biopsy. For instance, the c.613G > A variant (identified in identical twins in our study) still results in the production of a functional mRNA transcript: however, the substitution of serine for glycine at position 205 interferes with formation of the tetrameric PYGM structure. As a result, the misfolded protein either forms aggregates or is mis‐localised into the nucleus, resulting in rapid degradation and absence of detectable protein on Western blot.^41^

Measurement of muscle myophosphorylase activity (using a spectrophotometric assay that detects production of NADPH) has indicated that some genomic variants that could be considered ‘milder’ than others. Vissing and colleagues identified two intronic variants, c.425‐26A > G and c.856‐601G > A, that resulted in residual enzyme activity (1% and 2.5% of controls respectively).^41^ This correlated with improved peak workload and oxidative capacity, as well as improved generation of lactate during an ischaemic forearm test, when compared to patients with absent enzyme activity. Similarly, a patient compound heterozygous for a novel silent variant c.2430C > T, in conjunction the common variant c.148C > T, presented with minimal muscle symptoms, and myoglobinuria was detected only at the time of seizures.^22^ There was a trace of myophosphorylase staining suggesting at least some degree of residual enzyme activity, though muscle myophosphorylase activity was not performed.

Other genetic modifiers of the phenotype of GSD V have also been identified: specifically, a shorter (D) allele polymorphism of the angiotensin‐converting enzyme (*ACE*) gene is associated with a greater severity score, especially in patients who are homozygous for this polymorphism.^42^

As next generation sequencing for patients with neuromuscular symptoms becomes more commonplace, it is possible that more patients with milder genotypes and associated phenotypes will be recognised. However, accurate prediction of the phenotype based on genotype is still unlikely to be possible, given the substantial modulation of the clinical course by patterns of physical activity.

## 7 CONCLUSION

GSD V is a rare severe genetic metabolic disorder with a reported median diagnostic delay of 29 years. Hence there are sparse reports in children. Contrary to adults with this disorder second‐wind phenomenon is not clearly ascertained on history as children typically avoid triggering exercise. Genetic testing for GSD V should be considered in all cases of rhabdomyolysis in children, and in those who exhibit skeletal muscle related exercise limitation. Management involves the identification of the second wind and managing exercise and activities within those constraints. Patients may respond to exogenous glucose provision but this needs to managed carefully to avoid weight gain. Further research is required on optimal management of this disorder in childhood.

## AUTHOR CONTRIBUTIONS

Louisa Adams: first draft, data collection—input to subsequent drafts Ethics submission consent of families. Arthavan Selvanathan: literature review around genetic results and Table 1 data. Kiera J Batten: literature review around exercise testing. Nancy van Doorn: performed and designed all exercise tests apart from patient 7. Susan Thompson: management of patients—contribution to manuscript. Ashleigh Mitchell: management of patients—contribution to manuscript. Hugo Sampaio: diagnosis of patients—contribution to manuscript. Troy Dalkeith: management of patients—contribution to manuscript. Jacqui Russell: diagnosis and management of several patients. Carolyn J Ellaway: management of patients—contribution to several drafts. Michelle Farrar: diagnosis of patients—contribution to manuscript. Carolyn Broderick: supervision of all exercise tests apart from patient 7. Kaustuv Bhattacharya: concept, clinical management, analysis of data and production of graphs, paper guarantor, final draft.

## FUNDING INFORMATION

No specific funding was provided for this work.

## CONFLICT OF INTEREST STATEMENT

All authors declare no competing interest.

## ETHICS STATEMENT

Human Research Ethics Approval was granted by Sydney Children's Hospital Network (SCHN). Project reference CCR 2020/13. All children 18 years and under on 31st December 2020, managed at SCHN, with biallelic pathogenic variants of PYGM, were invited to participate.

## INFORMED CONSENT

Written informed consent was obtained from patients or their legal guardian.

## Data Availability

Data is stored on a secure public hospital server with no external access. The data can de‐identified and provided upon request.
